# Suspected phenobarbital-induced pancytopenia in a cat

**DOI:** 10.1177/2055116920916945

**Published:** 2020-05-27

**Authors:** Maria Lyraki, Helen Wilson

**Affiliations:** Bristol Veterinary School, University of Bristol, Langford, Bristol, UK

**Keywords:** Phenobarbital, pancytopenia, drug-induced reaction, anticonvulsants

## Abstract

**Case summary:**

A 3-year-old neutered male domestic shorthair cat developed pancytopenia 6 months after starting phenobarbital for treatment of recurrent seizures. The cat was switched from phenobarbital to levetiracetam and complete resolution of the pancytopenia was documented within 10 weeks, consistent with phenobarbital-induced pancytopenia.

**Relevance and novel information:**

While phenobarbital is frequently used as the first-line treatment for seizures in cats, phenobarbital-induced feline pancytopenia has not been documented in the veterinary literature before. Based on this case, regular monitoring of the complete blood count in cats receiving long-term phenobarbital treatment should be considered. In cases of persistent or severe haematological abnormalities, further investigations are required and treatment discontinuation may be needed in the absence of other causes of pancytopenia.

## Case description

A 3-year-old neutered male domestic shorthair cat was referred to our hospital with a 1-week history of hyporexia and lethargy. The primary care veterinary surgeon had performed a physical examination that revealed pale mucous membranes, an increased rectal temperature of 39.6°C (reference interval [RI] 37.5–39.5°C)^1^ and a neurological examination that was unremarkable. A complete blood count (IDEXX Laboratories) documented a poorly regenerative anaemia (haematocrit 21.6% [RI 28.2–52.7%], absolute reticulocyte count 76.6 × 10^9^/l [RI < 50.0 × 10^9^/l]),^2^ mature neutropenia (0.13 × 10^9^/l [RI 2.62–15.17 × 10^9^/l]) and thrombocytopenia (44 × 10^9^/l [RI 155–641 × 10^9^/l]). The cat was receiving phenobarbital (Epiphen; Vetoquinol) (3 mg/kg PO q12h) owing to a 6-month history of generalised tonic–clonic seizures suspected to be due to idiopathic epilepsy. Baseline investigations prior to starting phenobarbital treatment had been performed 6 months previously, including complete blood count, biochemistry, electrolytes, thyroxine, feline leukaemia virus (FeLV), feline immunodeficiency virus (FIV), coronavirus and *Toxoplasma gondii* serological testing, all of which were normal. The phenobarbital serum levels at the time of referral to our hospital were 25.9 mg/l (therapeutic RI 10–30 mg/l) and the most recent seizure episode was 2 months ago. No toxin or other drug exposure was reported and there was no travel history outside of the UK.

On presentation to our hospital, the cat was found to be quiet, but alert, with a persistently increased rectal temperature at 39.8°C (RI 37.5–39.5°C)^1^ and pale mucous membranes. Repeat complete blood count (Langford Vets Diagnostic Laboratories) documented a worsening regenerative anaemia (14.9% [RI 27.7–46.8%], absolute reticulocyte count 110 × 10^9^/l [RI < 50 × 10^9^/l]) thrombocytopenia (13 × 10^9^/l [RI 156–626 × 10^9^/l], estimate of three platelets per high power field [hpf]) and a persistent mature neutropenia (0.58 × 10^9^/l [RI 3–13.4 × 10^9/^l]) (Table 1; day 1). FeLV antigen, FIV antibodies and feline *Haemoplasma* species PCR in blood were all negative. Thoracic and abdominal ultrasound revealed no free fluid and there was no evidence of melaena or other external blood loss, therefore excluding haemorrhage as the cause of anaemia. The phenobarbital was discontinued on suspicion of a phenobarbital-induced blood dyscrasia and replaced by levetiracetam (20 mg/kg PO q8h) for the management of seizures. Treatment with doxycycline (10 mg/kg PO q24h) was initiated while waiting for outstanding results and discontinued after 7 days upon the receipt of negative feline *Haemoplasma* PCR testing. Furthermore, broad-spectrum antibiotic cover with amoxycillin/clavulanic acid (20 mg/kg PO q8h) was started in the light of the pyrexia and risk of sepsis associated with severe neutropenia. Further investigations including advanced imaging of the thorax and abdomen and bone marrow biopsy were offered but declined by the owner owing to financial constraints. Repeat complete blood count (Langford Vets Diagnostic Laboratories) 5 days after presentation to our hospital documented static regenerative anaemia (15.5% [RI 27.7–46.8%], absolute reticulocyte count 90 × 10^9^/l [RI < 50 × 10^9^/l]), worsened mature neutropenia (0.16 × 10^9^/l [RI 3–13.4 × 10^9^/l]) and improved thrombocytopenia (29 × 10^9^/l [RI 156–626 × 10^9^/l], an estimate of six platelets per hpf) (Table 1; day 5). The cat’s appetite and rectal temperature normalised within 6 days of starting treatment and the cat was discharged from our hospital with broad-spectrum antibiotic coverage with amoxycillin/clavulanic acid (20 mg/kg PO q8h) and anticonvulsant treatment with levetiracetam (20 mg/kg PO q8h).

**Table 1:** Serial monitoring of haematology in a cat with pancytopenia following phenobarbital discontinuation

	Day 1	Day 5	RI	Day 14	Day 42	Day 80	RI
HCT (%)	14.9	15.5	27.7–46.8	36.5	46.5	43.4	28.2–46.8
Absolute reticulocyte count (×10^9^/l)	110	90	<50	30	30	16.5	<50
Platelets (×10^9^/l)	13	29	156–626	100	111	140	155–641
Neutrophils (×10^9^/l)	0.58	0.16	3–13.4	0.43	4.45	2.93	2.62–15.17

RI = reference interval; HCT = haematocrit

On repeat examination 2 weeks later, the physical examination was normal and repeat complete blood count (IDEXX Laboratories) documented a normal haematocrit (36.5% [RI 28.2–52.7%], absolute reticulocyte count 30 × 10^9^/l [RI < 50.0 × 10^9^/l]), improved mature neutropenia (0.43 × 10^9^/l [RI 2.62–15.17 × 10^9^/l]) and improved thrombocytopenia (100 × 10^9^/l [RI 155–641 × 10^9^/l], an estimate of 3–8 platelets per hpf) (Table 1; day 14). Six weeks later, the physical examination was normal again and repeat complete blood count (IDEXX Laboratories) documented normal haematocrit (46.5% [RI 28.2–52.7%], absolute reticulocyte count 30 × 10^9^/l [<50.0 × 10^9^/l]), normal neutrophil count (4.45 × 10^9^/l [2.62–15.17 × 10^9^/l]) and improved thrombocytopenia (111 × 10^9^/l [155–641 × 10^9^/l], an estimate of eight platelets per hpf) (Table 1; day 42). The amoxicillin/clavulanic acid was discontinued at that point in the light of the normalised neutrophil count, which was considered to indicate the cat could now mount an adequate response to infection. A final re-examination 10 weeks after discharge documented one seizure since the last visit, but otherwise the cat was clinically well with normal physical examination and a normal complete blood count (IDEXX Laboratories): haematocrit 43.4% (RI 28.2–52.7%), absolute reticulocyte count 16.5 × 10^9^/l (RI < 50.0 × 10^9^/l), neutrophil count 2.93 × 10^9^/l (RI 2.62–15.17 × 10^9^/l] and platelet count 140 × 10^9^/l (RI 155–641 × 10^9^/l) and an estimate of 10 platelets per hpf (Table 1; day 80).

## Discussion

Pancytopenia is defined as the combination of anaemia, neutropenia and thrombocytopenia, and is indicative of bone marrow suppression.^3^ Previously reported causes of pancytopenia in cats include infectious agents, immune-mediated diseases, myelofibrosis, neoplasia, and toxin-induced and drug-induced pancytopenia.^4^ The drugs that have been previously associated with pancytopenia in cats are griseofulvin,^5^ albendazole,^6^ azathioprine^7^ and doxorubicin.^8^ To our knowledge, phenobarbital has not previously been reported to cause pancytopenia in cats, although it has been previously documented in dogs.^9–13^ We report a cat on long-term phenobarbital treatment that developed marked pancytopenia, which resolved after phenobarbital discontinuation.

Phenobarbital is a barbiturate commonly recommended as a first-line drug for cats with recurrent seizures.^14–18^ The drug-related adverse effects can be divided into type A and type B.^19^ Type A includes the adverse effects that can be explained by the known pharmacological properties of the agent and therefore are usually dose dependent and predictable. Type B adverse reactions are independent from the known mechanism of action of the drug and the drug dose and are therefore unpredictable.^19^ Type A adverse effects of phenobarbital reported in cats include sedation, pelvic limb ataxia, paraparesis, polyphagia, polydipsia, weight loss, increased alanine transaminase and alkaline phosphatase activities, and behavioural changes.^20^ Type B adverse effects reported in cats include dermatitis, pruritus, stomatitis, hypersensitivity,^21^ pseudolymphoma,^22^ fever,^23^ coagulopathies, leukopenia and thrombocytopenia.^15^ According to the existing literature, haematological abnormalities were mild, transient and did not cause a treatment discontinuation.^20^ In our case, the severe pancytopenia and associated hyporexia and fever required hospitalisation, phenobarbital discontinuation and broad-spectrum antibiotic coverage. In one recent study regarding the follow-up of cats on phenobarbital treatment, the adverse effects tended to be more severe at the beginning of treatment and at higher doses, although haematological adverse effects were uncommon and the timing of these was not specifically described.^20^ In dogs, the time to onset of pancytopenia has been reported to be most common within the first 3 months after initiation of phenobarbital treatment.^19^ In our case report, the cat developed pancytopenia after 6 months of treatment and in spite of the fact that the phenobarbital dose was within the recommended range, suggesting that not only short-term, but also long-term, regular monitoring of the complete blood count is recommended.

The exact mechanism of phenobarbital-induced pancytopenia is not well clarified,^24^ although both myelofibrosis and bone marrow necrosis have been documented in dogs receiving phenobarbital treatment.^13,25^ The proposed mechanisms extrapolated from human medicine are either an immune-mediated destruction of the precursor cells in the bone marrow or direct toxic effects of the drug on the bone marrow.^26^ Phenobarbital has been reported to induce cytochrome P450 enzymes in dogs, which can enhance the drug metabolism of phenobarbital itself.^12,27,28^ Interestingly, phenobarbital does not induce these enzymes in cats,^12^ which could theoretically lead to higher plasma concentrations and therefore more likely adverse drug effects. However, this is not reflected in the frequency of reported cases in the literature of adverse effects associated with phenobarbital in cats and the cat reported here had a measurably normal serum phenobarbital concentration.

In our case report, levetiracetam was selected to replace the phenobarbital monotherapy treatment. A recent systematic review in feline antiepileptic drugs found overall weak evidence to support the safety and efficacy of different anticonvulsant options.^15^ Despite that, the results suggested levetiracetam to be the next most effective treatment after phenobarbital.^15^

A few limitations apply to this case report, mainly owing to cost constraints. First, bone marrow biopsy and advanced thoracic and abdominal imaging would be required to fully rule out other causes of pancytopenia. A novel bone marrow aspiration technique via a costochondral rib using local anaesthesia alone has been proposed in one case report of a dog and may be useful in patients too unstable to undergo general anaesthetic,^29^ although the safety of such a technique has not been demonstrated in cats. However, bone marrow aspiration was declined on financial grounds in this cat. Nevertheless, the resolution of the cat’s pancytopenia following discontinuation of phenobarbital, in the absence of other treatment modalities for pancytopenia, indicates that phenobarbital was the cause. Furthermore, the cat was presumed to have idiopathic epilepsy based on the clinical response to anticonvulsants and the unremarkable blood testing but advanced brain imaging, cerebrospinal fluid analysis and liver function testing would, ideally, have been performed to rule out underlying diseases. Nonetheless, this had no influence on the fact that the phenobarbital appears to have caused the pancytopenia in this case.

## Conclusions

To our knowledge, this is the first report of suspected phenobarbital-induced pancytopenia in a cat. Considering that this possible adverse effect may be life-threatening, regular ongoing monitoring of the complete blood count of all cats treated with phenobarbital should be considered. If any haematological abnormalities identified are persistent or severe, further investigations are required and phenobarbital discontinuation may be needed in the absence of other causes of pancytopenia.
